# Diagnosis and Management of Hyponatremia in Patients with Aneurysmal Subarachnoid Hemorrhage

**DOI:** 10.3390/jcm4040756

**Published:** 2015-04-21

**Authors:** Neena I. Marupudi, Sandeep Mittal

**Affiliations:** Department of Neurosurgery, Wayne State University, Detroit Medical Center, 4160 John R Street, Suite 930, Detroit, MI 48201, USA; E-Mail: nmarupud@med.wayne.edu

**Keywords:** hyponatremia, subarachnoid hemorrhage, SIADH, cortisol insufficiency, sodium

## Abstract

Hyponatremia is the most common, clinically-significant electrolyte abnormality seen in patients with aneurysmal subarachnoid hemorrhage. Controversy continues to exist regarding both the cause and treatment of hyponatremia in this patient population. Lack of timely diagnosis and/or providing inadequate or inappropriate treatment can increase the risk of morbidity and mortality. We review recent literature on hyponatremia in subarachnoid hemorrhage and present currently recommended protocols for diagnosis and management.

## 1. Introduction

**Figure 1:**
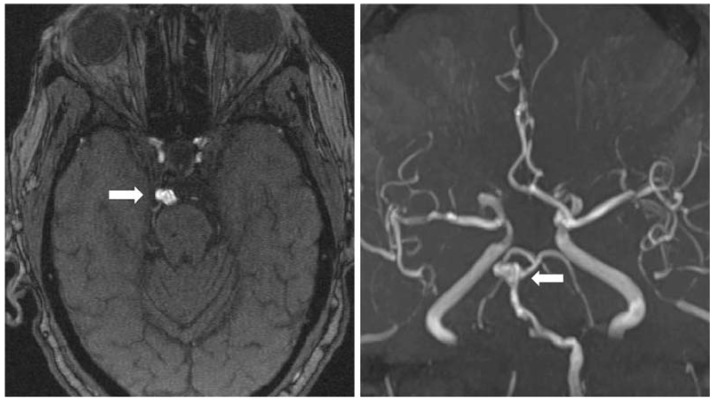
Magnetic resonance angiography of a 79-year-old woman with fusiform aneurysm of the basilar artery apex measuring 9.3 × 5.0 × 7.0 mm in transverse, anteroposterior and cranio-caudal dimensions, respectively (arrows).

**Figure 2:**
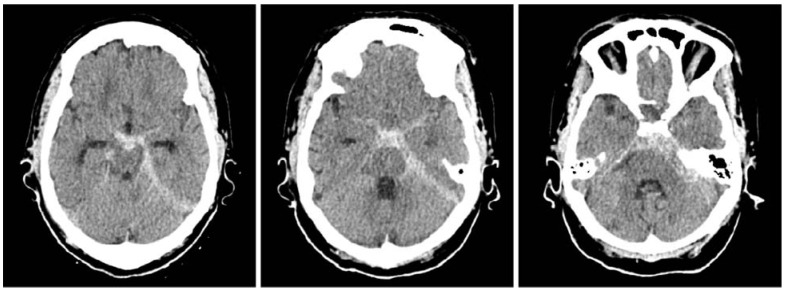
A 79-year-old woman with known basilar tip aneurysm presenting with acute onset of severe headache (same patient as in Figure 1). The non-contrast CT scan study demonstrates diffuse subarachnoid hemorrhage in the perimesencephalic cistern and left ambient cistern extending along the left tentorium, consistent with rupture of basilar tip aneurysm.

**Figure 3:**
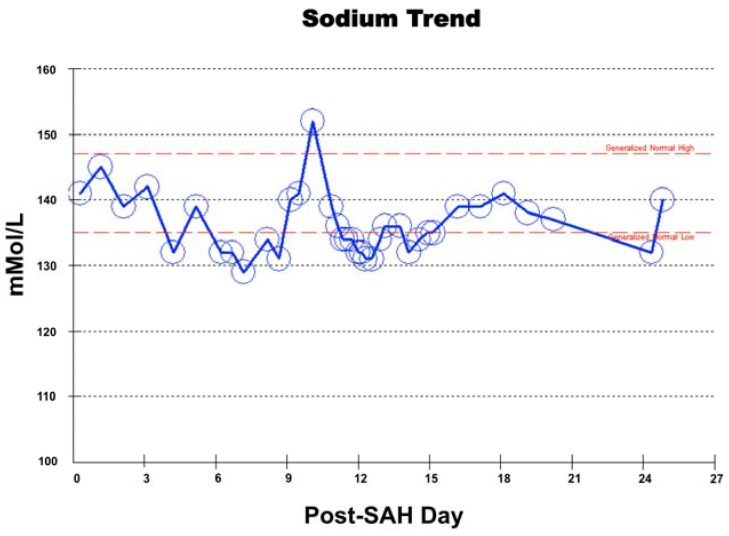
Serum sodium levels following subarachnoid hemorrhage (SAH) from presentation through 3.5 weeks post-hemorrhage in a 79-year-old woman with rupture of fusiform basilar tip aneurysm (same patient as in Figure 1 and Figure 2).

The most common electrolyte abnormality that develops in patients with subarachnoid hemorrhage (SAH) is hyponatremia and has a prevalence ranging from 30%–56% [1,2,3]. The most common non-traumatic or spontaneous cause of SAH is a ruptured cerebral aneurysm (Figure 1), resulting in extravasation of blood into the space between the pia and arachnoid mater (Figure 2). Although previously defined as a serum sodium concentration level of less than 135 mEq/L, severe or significant hyponatremia has recently been redefined as serum sodium less than 131 mEq/L [4]. Hyponatremia following SAH is most commonly associated with syndrome of inappropriate antidiuretic hormone secretion (SIADH). Other associated causes include acute cortisol insufficiency, cerebral salt wasting syndrome (CSW), excessive fluid therapy and/or diuretic therapy. Regardless of the underlying etiology, hyponatremia in SAH patients is associated with a longer hospital course, increased morbidity and risk of vasospasm [2]. Hyponatremia post-SAH can develop within a few days to within a few weeks and be associated with drastic fluctuations in sodium levels with treatment (Figure 3). Because hyponatremia is associated with a poor outcome in patients with SAH, anticipation, timely detection and appropriate treatment are necessary to improve patient outcomes. In this article, we review recent outcome studies and discuss current trends for underlying causes, risk factors, clinical implications and treatment of hyponatremia in patients following SAH.

## 2. Causes of Hyponatremia

In most studies, SIADH was categorized as the most frequent cause of severe hyponatremia among patients with aneurysmal SAH [5,6]. In SIADH, excessive secretion of antidiuretic hormone is caused by stimulation of the hypothalamus with various traumatic or ischemic factors, resulting in the enhancement of water reabsorption in the distal convoluted tubule of the kidney, causing fluid retention and dilutional hyponatremia. Nonetheless, the etiology of hyponatremia after SAH is diverse. In addition to SIADH, CSW, acute cortisol insufficiency, excessive intravenous fluid therapy, diuretic therapy or a combination of these entities may contribute to increased natriuresis. Appropriate therapy must be targeted for the correct etiology of hyponatremia to be effective in diminishing morbidity or mortality associated with hyponatremia in SAH patients.

In CSW, the secretion of antidiuretic hormone (ADH) in the plasma is normal, but is characterized by urinary sodium excretion, as well as reductions of extracellular fluid and circulating blood volume, causing hyponatremia. CSW is the cause of hyponatremia in only a minority of cases [1,7,8]. Kao *et al.* reported 34.5% of severe hyponatremia to be due to SIADH, while 23% were considered to be due to CSW [5]. Of note, the patients included in this study had more severe SAH than in the comparative studies, and the inclusion criterion was a plasma Na <130 mEq/L.

Although there is considerable dispute regarding the incidence of the various etiologies of hyponatremia in SAH, SIADH has clearly been established as the most common cause of hyponatremia post-SAH. In patients with CSW, an abnormal release of natriuretic hormones may contribute to the excessive natriuresis. Previous studies supporting a higher incidence of CSW in SAH investigated plasma concentrations of atrial natriuretic peptide (ANP) and brain natriuretic peptide (BNP) concentrations, which rise after SAH [9,10,11]. More recent data suggest that elevated BNP concentrations do not mediate the development of hyponatremia [12]. The separation between SIADH and CSW may not be clear in pathologies, such as SAH. Multiple abnormalities occurring in combination, including an increase in natriuretic peptide secretion, an inhibition of the renin-aldosterone system and excessive adrenergic activity, may contribute to hyponatremia [13].

Unlike SIADH and CSW, the incidence of electrolyte imbalance as a result of cortisol deficiency has not been well investigated in SAH patients. Routine examination of adrenocorticotropic hormone (ACTH)/cortisol dynamics is not typically part of SAH work-up and management. Klose *et al.* and Parenti *et al.* investigated pituitary function post-SAH and found that between 7.1% and 12% of patients are cortisol-deficient at the time of presentation with SAH [14,15]. Acute cortisol deficiency may still be underestimated in the SAH patient population, as is also seen in the traumatic brain injury patient population [16]. A transient cortisol deficiency may easily be missed with a single cortisol measurement due to the usual physiologic fluctuations of plasma cortisol levels through the course of a day.

Based on specific clinical assessment protocols, the current suggested etiologies for hyponatremia in SAH include SIADH in over 70% of patients, followed by incorrect or injudicious intravenous fluid use (e.g., administration of 0.45% saline) in 10%, volume depletion in 10% and acute cortisol deficiency in 8% [17]. In the first study to evaluate vasopressin levels in hyponatremic SAH patients, Hannon *et al.* found that in the SIADH group of SAH patients, ADH was significantly higher before and during the hyponatremic period compared with ADH levels measured once hyponatremia had resolved [17]. Moreover, urine volumes fell as plasma ADH concentration rose in the group, a pattern suggesting a causative role for ADH in the development of hyponatremia and supporting the diagnosis of SIADH. ADH levels were suppressed in patients with dilutional hyponatremia, and ADH concentrations were also lower in the ACTH-deficient and volume-depleted groups in comparison to the SIADH group [17]. Other peptides released after SAH, such as adrenomedullin and endothelin, possess vasoconstrictive or vasodilatory properties [18,19]. These peptides and their association with other natriuretic peptides may explain the suggested link between hyponatremia and increased risk of cerebral vasospasm [18,19].

Overall, the most common cause of hyponatremia from aneurysmal SAH is clearly SIADH, although acute cortisol deficiency does account for a small, but clinically important fraction of cases of hyponatremia.

## 3. Presentation, Incidence and Risk Factors

Up to one-half of patients suffering from aneurysmal SAH develop hyponatremia during their clinical course [1,2,3]. Neurological disease processes and the postoperative period are established risk factors for hyponatremia and contribute to the high prevalence of hyponatremia seen post-SAH [20,21]. A recent article investigating the outcomes of hyponatremia in 59 aneurysmal SAH patients determined a prevalence of 37% and a mean lowest sodium level of 126.97 mEq/L for a median duration of two days [22]. Frequently observed during the acute phase after aneurysmal SAH, hyponatremia following SAH may be more common in patients who are more than 50 years old [22].

Where serum sodium values are from 115–120 mEq/L, signs and symptoms associated with hyponatremia can include fever, headache, nausea and vomiting, muscle cramps, muscle weakness and confusion [23]. With values less than 110 mEq/L, more severe neurological symptoms of stupor, seizures and coma can occur [23]. Sudden decreases in serum sodium elicit more severe symptoms than gradual decreases that occur over days to weeks.

A difference seen between various retrospective studies is the time period for the development of hyponatremia after SAH. Hyponatremia developed later in the hospital course, at about seven days post-SAH in some studies, while other recent cohort studies identified hyponatremia early in the hospital course [1,5,7]. Hannon *et al.* noted in their series of 100 patients that 73.4% of hyponatremic SAH patients developed hyponatremia between post-hemorrhage Days 1 and 3, that 12.2% developed it between Days 4 and 7 after SAH and that 14.3% developed hyponatremia more than seven days after SAH [17]. No correlation could be drawn between the severity/grade (measured by either Hunt and Hess scale or Fisher grade) of SAH and the development of hyponatremia.

The location of aneurysmal rupture may be associated with the incidence of post-SAH hyponatremia. Hyponatremia was seen in 52.4% of patients with anterior communicating artery (AComA) aneurysms, followed by 33.3% of patients with middle cerebral artery and posterior communicating artery aneurysms and only 27.7% of patients with internal carotid artery aneurysms and multiple aneurysms [22]. The rupture of an AComA aneurysm may be associated with a higher incidence of hyponatremia, because the hypothalamus is supplied by perforating vessel branches of the AComA. When associated vasospasm in this area occurs, hypothalamic ischemic damage is caused, thereby resulting in hormone secretion abnormalities and hyponatremia [24].

A larger series, however, did not find any significant difference in the incidence of hyponatremia and the anatomic territory of the ruptured aneurysm [17]. No difference in the incidence of hyponatremia was seen between those patients who had craniotomy and clipping of the cerebral aneurysm *versus* endovascular coiling, and no significant difference was noted between patients that had intervention and those that did not [17].

## 4. Clinical Implications

Hyponatremia after SAH may be an independent risk factor for poor outcome [25,26]. Hyponatremia and associated hypotonicity result in a shift of water from the extracellular to intracellular space and can worsen cerebral edema and intracranial hypertension in patients with SAH, further increasing the risk of seizures and neurological injury [27].

Uncontrolled prospective studies suggest a relationship between hyponatremia and excessive natriuresis and volume contraction [28,29]. Volume contraction has been shown to be associated with symptomatic vasospasm [30], and hyponatremia and fluid restriction are related to an increased incidence of delayed ischemic deficits [25,29]. Of note, in a cohort study of 580 patients with SAH, hyponatremia was less frequently associated with poor outcome than hypernatremia [31]. Because SAH patients frequently require therapeutic infusion of hyperosmolar fluids to help mitigate intracranial hypertension, the resultant hypernatremia is linked with poor outcomes in this patient population. Currently, normonatremia is considered the conservative, but safe management strategy for SAH patients.

## 5. Treatment and Management

**Figure 4:**
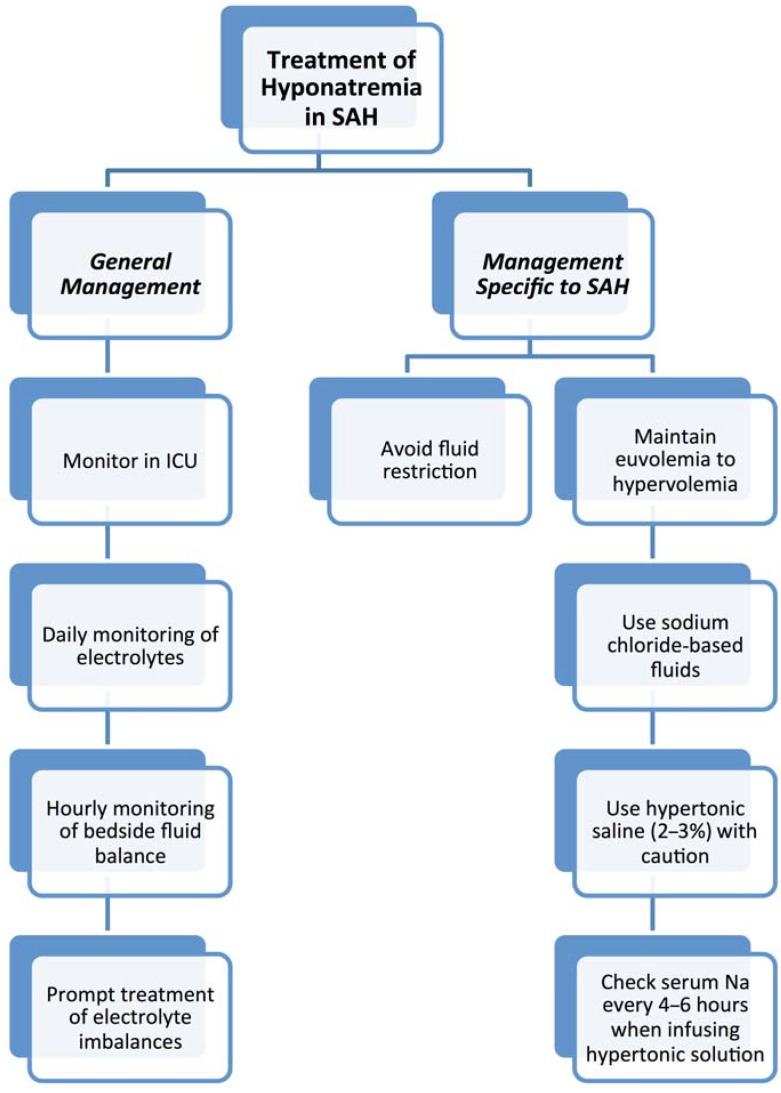
Treatment algorithm of hyponatremia in SAH.

Determination of the cause of hyponatremia from clinical examination and biochemical hormone measurement is essential to ensure timely and effective treatment of the hyponatremia. Patients with aneurysmal SAH are monitored in an intensive care setting, preferably with specialized neurointensive care, for at least 14 to 21 days post-SAH, to allow for close clinical monitoring of signs and symptoms of cerebral vasospasm. Along with vasospasm monitoring, comprehensive monitoring of electrolytes and fluid balance on a daily basis ensures early detection and efficient management of hyponatremia after SAH, thereby reducing morbidity and mortality. A daily check of electrolytes is typical and should be routine. Patients who require intravenous hypertonic therapy require more frequent sodium checks (every 4 to 6 h). Any changes in mental status, large fluctuations of fluid balance and/or polyuria should prompt urgent investigation of serum sodium levels (Figure 4).

Correcting hyponatremia too rapidly can result in central pontine myelinolysis; on the other hand, insufficient correction of hyponatremia can result in cerebral edema, seizures, coma or death. Even mild hyponatremia is associated with increased mortality [32]. Fluid restriction to correct hyponatremia is potentially more deleterious in patients with SAH with possible associated increased risk of cerebral vasospasm [11,33]. When hyponatremia is treated promptly and appropriately, patients’ sodium levels are normalized without deleterious effects.

Audibert *et al.* assessed the endocrinological response to severe SAH and found changes in plasma concentration of several hormones: ADH, aldosterone, renin, angiotensin, ANP and BNP [34]. Although changes in these hormone levels are noted during the first 12 days post-SAH, it is not practical to rapidly and accurately obtain hormone measurements, because their profile changes frequently. The authors suggested that measuring bedside fluid and sodium balance is the most valuable and cost-effective method for preventing and managing hyponatremia in patients with SAH [34].

Traditionally, patients with cerebral insults, like SAH, are maintained on sodium chloride-based fluids (*i.e.*, 0.9% saline) for baseline and fluid replacement requirements. Such intravenous solutions avoid cerebral edema caused by fluid shifts across a damaged blood-brain barrier [33]. The current guidelines of the Neurocritical Care Society for the management of patients with SAH recommend avoiding large amounts of free water intake and avoiding fluid restriction to treat hyponatremia [35]. In addition, the guidelines of the American Heart Association recommend that volume contraction be treated with isotonic fluids (Class IIa, Level B evidence) and that large volumes of hypotonic fluids should generally be avoided in patients with SAH [36]. The guidelines, however, did not address or make recommendations on the composition of baseline fluid administration in SAH patients. Lehmann *et al.* recently published the results of a small, randomized study suggesting that administration of balanced crystalloids and colloid solutions (those with electrolyte compositions similar to plasma) in SAH patients did not result in more frequent hyponatremia or hypo-osmolality [37]. Balanced solutions in the early SAH period may prevent electrolyte imbalance associated with saline-based intravenous fluids, such as hyperchloremia, hyperosmolality and extreme positive fluid balances [37].

SIADH is best treated by fluid restriction to less than 500 mL/day; however, such treatment may not be feasible in SAH patients, because many of these patients are not fully conscious and require enteral tube feeding, resulting in a daily fluid intake of 1–2 L. In addition, hypovolemia from fluid restriction is associated with an increased risk of vasospasm and subsequent cerebral ischemia [36]. A number of alternative therapies have been tried, but overall efficacy still remains somewhat controversial without randomized clinical control trials. Therapeutic options for water restriction include hypertonic saline solutions and albumin [38,39]. Hypertonic saline, usually administered as a 2% or 3% solution, can increase plasma sodium concentration efficiently and very rapidly, but it also increases blood volume and the risk of pulmonary edema and heart failure; associated neurological complications are suspected, but further investigation regarding the overall safety profile is necessary. The effects of hypertonic saline are generally transient, because the physiological stimuli for water retention and secondary natriuresis remain present. The effects of albumin in limiting natriuresis have been reported in a few trials, but its effectiveness remains controversial [40,41]. Fludrocortisone may enhance sodium retention through its mineralocorticoid properties, but studies suggest that its ability to correct hyponatremia is limited and is associated with complications of fluid overload [42,43]. Alternatively, vasopressin receptor antagonists (e.g., conivaptan) have been proposed and trialed in small studies, but have not become routine therapy, due to the potential for harmful, rapid and drastic changes in plasma sodium [44,45]. In one study, a single dose of conivaptan increased plasma sodium by at least 4 mEq/L in most patients and maintained the sodium improvement for three days [44]. Another study using conivaptan demonstrated increased plasma sodium of 6 mEq/L with maintenance of the effects for an average of 13 h [45]. In both studies, the number of SAH patients was only 12 patients, and patients were simultaneously treated with fludrocortisone or hypertonic saline in some cases. Conivaptan’s efficacy and safety profile remain controversial, because one-third of the patients in the study proceeded to become hyponatremic again after its discontinuation, and both studies limited the administration of the drug to a single bolus. Another therapeutic option under recent investigation is urea [46]. Urea acts by inducing osmotic water elimination and by promoting passive sodium resorption in the ascending limb of the loop of Henle of the nephron [39].

Acute cortisol deficiency typically resolves after the administration of parenteral hydrocortisone. Still, the impact of low-dose corticosteroid therapy still requires further investigation in this population for two reasons [47]. First, it is not clear whether acute relative adrenal insufficiency after aneurysmal SAH is responsive to corticosteroid therapy or whether resolution of the cortisol deficiency is related to the expected clinical course of adrenal insufficiency post-SAH. Secondly, treatment with corticosteroids, theoretically, could have a role in the management of cerebral vasospasm in these patients. Natriuresis after SAH induces osmotic diuresis and decreases blood volume, increasing the risk of cerebral vasospasm. A randomized controlled trial of hydrocortisone in patients with SAH demonstrated that treatment with hydrocortisone prevented excess natriuresis and potentially decreased the incidence of cerebral vasospasm; however, the study did not demonstrate a statistically significant difference in the incidence of symptomatic cerebral vasospasm or in overall outcome [48].

Overall, the management of hyponatremia in SAH patients requires further investigation of treatment options that avoid fluid restriction, and additional studies will help standardize optimal care.

## 6. Outcomes

Hyponatremia has been associated with an increased duration of hospitalization in some studies [5,6]. Other studies did not find such an association [17], but rather, found an association with a longer length of stay in the intensive care unit [22]. Reported rates of poor outcome, based on the Glasgow Outcome Score, either severe disability or death, is 22%–32% in hyponatremic patients with aneurysmal SAH [3,22]. In fact, patients with aneurysmal SAH with hyponatremia are 15-times more likely to have a poor outcome [3] compared to their normonatremic counterparts.

## 7. Conclusions

Hyponatremia is seen in a considerable number (30%–56%) of patients with aneurysmal SAH. Although it is a readily diagnosable and treatable condition, patient morbidity is significantly increased in this population. The etiology of hyponatremia after aneurysmal SAH remains controversial, but likely stems from multiple causes, including SIADH, fluid imbalances and acute cortisol insufficiency. Adequate understanding of the underlying etiology of hyponatremia is essential for safe, effective and timely management of hyponatremia following aneurysmal SAH.
